# The Retroperitoneal Laparoscopic Renal Capsulectomy for Spontaneous Renal Subcapsular Fluid Collection: A Case-Series Report and Literature Review

**DOI:** 10.1097/MD.0000000000003751

**Published:** 2016-05-27

**Authors:** Guodong Zhu, Dapeng Wu, Kaijie Wu, Wenbin Song, Zhishang Yang, Yue Zhang, Linlin Zhang, Dalin He

**Affiliations:** From the Department of Urology, The First Affiliated Hospital of Xi’an Jiaotong University, Xi’an, P. R. of China.

## Abstract

Spontaneous renal subcapsular fluid collection may occur as a rare presentation of nephritic syndrome, and distension of the renal capsula and Gerota fascia due to massive fluid accumulation may cause pain. In addition, hypertension secondary to renal ischemia and activation of renin–angiotensin–aldosterone system may also occur. The objective of this study is to evaluate the surgical outcome of retroperitoneal laparoscopic renal capsulectomy for patients with this disease.

We retrospectively analyzed the clinical data of 10 female patients with spontaneous renal subcapsular fluid collection, diagnosed with B ultrasound and enhanced computed tomography (CT) scan. Eight patients first underwent percutaneous renal subcapsular drainage, which seemed to be less effective, and then all patients underwent retroperitoneal laparoscopic renal capsulectomy. The volume of renal subcapsular fluid was documented, the fluid was examined by routine biochemical tests, and the excised renal capsules underwent pathological examination individually. The postoperative drainage time for each patient was documented, and follow-up was conducted 1, 3, 6, 12 months, and 2 years postoperatively.

Retroperitoneal laparoscopic renal capsulectomy was successfully performed in all patients with no major complications. The average volume of renal subcapsular fluid was 436 milliliter (mL, 180–880 mL) in light yellow color, and the concentration of creatinine and urea nitrogen was quite similar to that of serum. The pathological findings revealed fibrous dysplasia of the renal capsule with chronic infiltration of inflammatory cells. The average drainage time was 11.5 days (5–30 days) postoperatively. All patients recovered 1 month after the operation and there were no recurrences with a mean follow-up period of 12 months (6–24 months).

The reason for spontaneous renal subcapsular fluid collection is unknown, and the aim of treatment is mainly to alleviate symptoms. In our experience, retroperitoneal laparoscopic renal capsulectomy is an effective surgical treatment, especially for patients who were refractory to percutaneous renal subcapsular drainage, with no observed recurrence.

## INTRODUCTION

The renal capsula is a fibrous membrane, which surrounds the kidney parenchyma and extends to the upper ureter, and it is very easy to separate from the renal parenchyma. So, any hemorrhage, inflammatory exudation, urine infiltration, or lymphatic fluid from the kidney parenchyma can be easily accumulated under the renal capsula and form the renal subcapsular fluid collection, which is usually associated with spontaneous or secondary lesions from the kidney or perinephric spaces.^1^ The secondary renal subcapsular fluid collection is more commonly seen, and may be caused by flank blunt trauma, renal benign or malignant tumor, kidney parenchyma infection, acute upper urinary tract obstruction, renal vascular diseases, and some iatrogenic manipulations, like extracorporeal short wave lithotripsy and ureteroscopy.^2^ However, the spontaneous renal subcapsular fluid collection is very rare, and caused by unknown reasons. The patients may complain flank or abdominal pain, due to the distension of the renal capsula or Gerota fascia with increased pressure caused by the massive accumulated fluid. In addition, the patients may have secondary hypertension, which is caused by renal ischemia and activation of the renin–angiotensin–aldosterone system. So, prompt diagnosis and treatment is essential.^3^ In this study, we share our experience with diagnosis and retroperitoneal laparoscopic renal capsulectomy for patients with spontaneous renal subcapsular fluid collection, and evaluated the surgical outcomes for these patients.

## METHODS

### Patients and Clinical Data

Ten female patients were diagnosed with spontaneous renal subcapsular fluid collection at our institution between January 2010 and December 2012. The study protocol was reviewed and approved by the Institutional Review Board of The First Affiliated Hospital of Xi’an Jiaotong University. The mean age of the patients was 42.3 years (22–65 years). One patient had a history of hypertension, and the other 9 patients had no special history. All the patients had flank pain or discomfort with no history of trauma or iatrogenic injuries, and were diagnosed with renal subcapsular fluid collection by abdominal B ultrasound or computed tomography (CT) scan.

Eight patients first underwent percutaneous renal subcapsular puncture, drainage, and alcohol injection, but it seemed to be less effective. To be specific, after percutaneous drainage, the daily drainage volume of 6 patients was above 500 milliliter (mL) and lasting for more than 2 weeks, with no obvious symptom relief in 2 of them. The other 2 patients had recurrent renal subcapsular fluid collection identified by ultrasonography with lumbar pain, after 4 and 6 months of the percutaneous drainage, respectively.

A contrast CT scan of the kidney was performed for each patient after they were admitted to our institution, and these clearly showed renal subcapsular fluid collection with an intact renal capsula and enhanced renal parenchyma. They were diagnosed with spontaneous renal subcapsular fluid collection, and all received retroperitoneal laparoscopic renal capsulectomy under general anesthesia. The volume of renal subcapsular fluid for each patient was documented, the fluid was examined by routine biochemical tests, and the excised renal capsules underwent pathological examination. The time period for drainage and any complications after the operation were documented for each patient. Follow-up was conducted 1, 3, 6, 12 months, and 2 years after the operation by abdominal B ultrasound and CT scan.

### Surgical Procedure

The patient was placed in a healthy lateral decubitus position with a 2-centimeter (cm) incision below the 12th rib in the posterior axillary line, and then the musculature and lumbodorsal fascia were separated bluntly by a straight blood-vessel forcep. The peritoneum was pushed away by the middle finger in the retroperitoneal space, into which a self-made balloon was placed, and about 600–1000 mL air was infused into the balloon to expand the retroperitoneal space for 3 minutes (min). With the guidance of the middle finger in the expanded retroperitoneal space, a 1 cm skin incision was made about 2 cm above the spina iliace in the midaxillary line, and then a 10-millimeter (mm) trocar was placed into it, through which the laparoscopic endoscope was positioned. Under the guidance of the endoscope, a small skin incision was made below the costal arch in the anterior axillary line with a 12 mm trocar was placed, and another 12 mm trocar was placed into the incision in the posterior axillary line. A carbon dioxide pneumoretroperitoneum of 10–13 mm of mercury was maintained, and the surgical instruments were placed into trocars in the anterior and posterior axillary line, respectively. A laparoscopic hook electrode or laparoscopic ultrasonic dissector was inserted as needed. A combination of blunt and sharp dissection of the Gerota fascia and perirenal fat was used until the domed renal capsula could be recognized. Then, a laparoscopic puncture needle suction was inserted into the renal subcapsular cavity, and the collected fluid was aspirated. Finally, the thickened renal capsula was incised with a laparoscopic hook electrode or a laparoscopic ultrasonic dissector up to the limit of the normal renal parenchyma edges. The renal subcapsula cavity was thoroughly examined to exclude the presence of possible neoplastic change. A 24 French closed drain was left in the retroperitoneal space through the trocar in the midaxillary line with the suturing of the skin incisions. The entire renal capsula was subjected to histopathological examination, and the renal subcapsular fluid was subjected to biochemical analysis.

### Literature Search

We conducted a systematic search of the PubMed online database for published original articles or case reports from January 1995 to December 2015 using the terms “renal subcapcular fluid,” “perirenal fluid,” or “perinephric fluid” to make the literature retrieval. Only those studies that reported patients with renal subcapcular fluid collection could be involved in our further analysis in the discussion part. From each involved article, we extracted the first author's last name, publication year, country, the number and gender of patients, the causes for kidney subcapcular fluid collection, and the management for the patients or outcomes. All these data were summarized in Table 1 and analyzed in the discussion part.

**TABLE 1:**
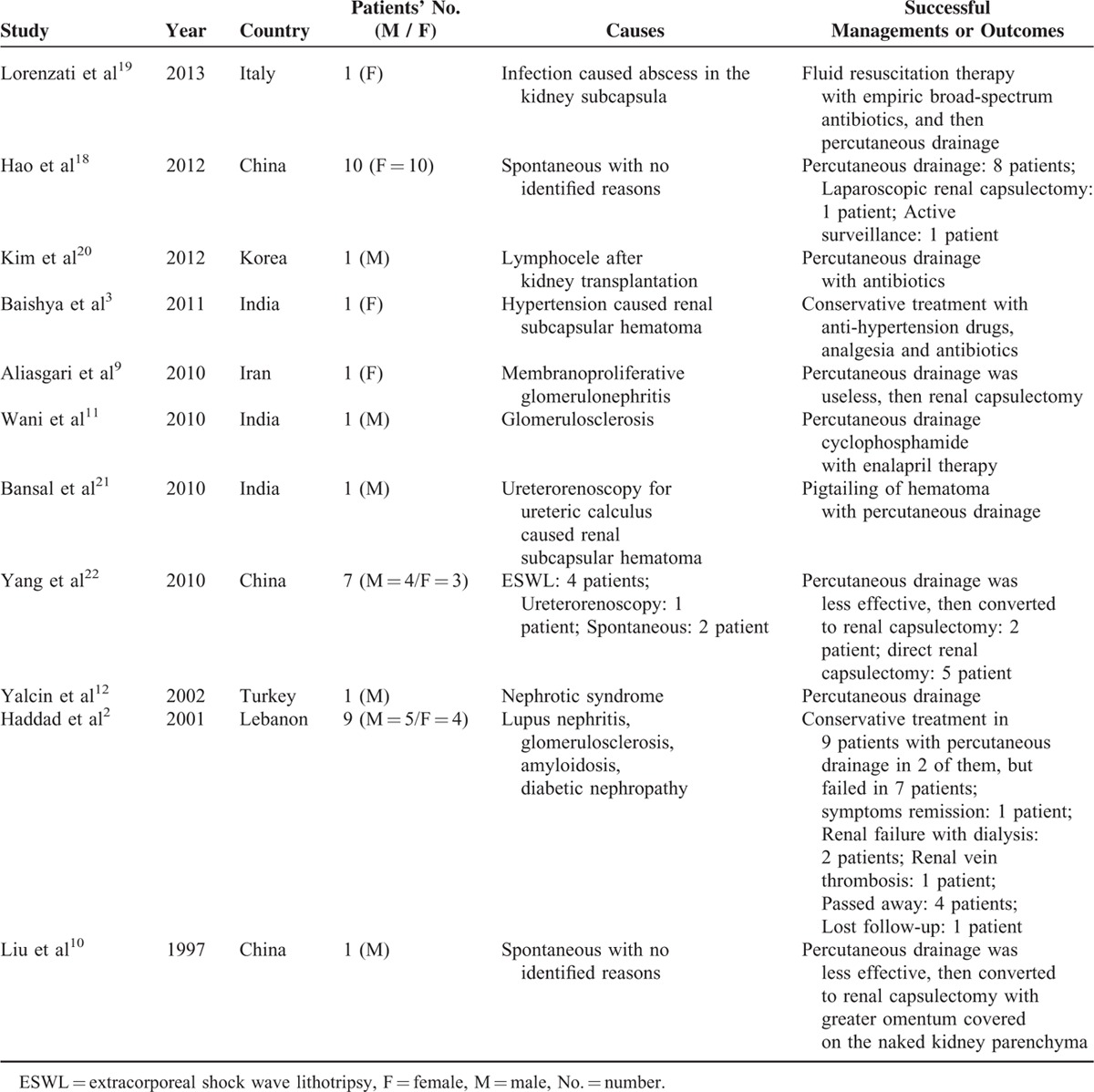
Summary of the Key Data of the Main Publications for Patients With Renal Subcapsular Fluid Collection

## RESULTS

**FIGURE 1:**
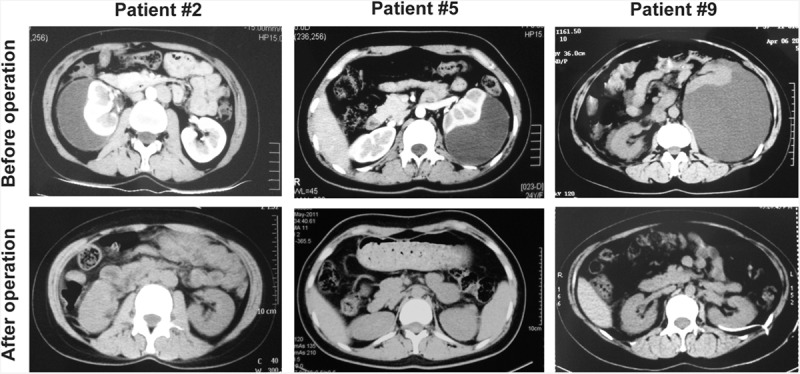
CT scan images before and after retroperitoneal laparoscopic renal capsulectomy for 3 individual patients with spontaneous renal subcapsular fluid collection. The contrast CT scan for patient #2 showed spontaneous renal subcapsular fluid collection with the maximum length of 9.0 cm in the right kidney, which largely regressed 1 month after the operation as demonstrated by CT scan. Patient #5 had spontaneous renal subcapsular fluid collection with a maximum length of 10.0 cm in the left kidney, 3 months after surgery, CT scans showed complete clinical regression. Patient #9 had spontaneous renal subcapsular fluid collection in both kidneys with a notably larger diameter of 15.5 cm on the left side; 6 months after retroperitoneal laparoscopic left renal capsulectomy, fluid collection almost disappeared in the left kidney. CT = computed tomography.

Spontaneous renal subcapsular fluid collection was demonstrated by contrast CT scan of the kidneys, revealing a mean maximum length of 11.8 cm (7.5–20.5 cm, Table 2) before retroperitoneal laparoscopic renal capsulectomy. Renal subcapsular fluid collection almost disappeared in follow-up CT scans after the operation (Figure 1).

**TABLE 2:**
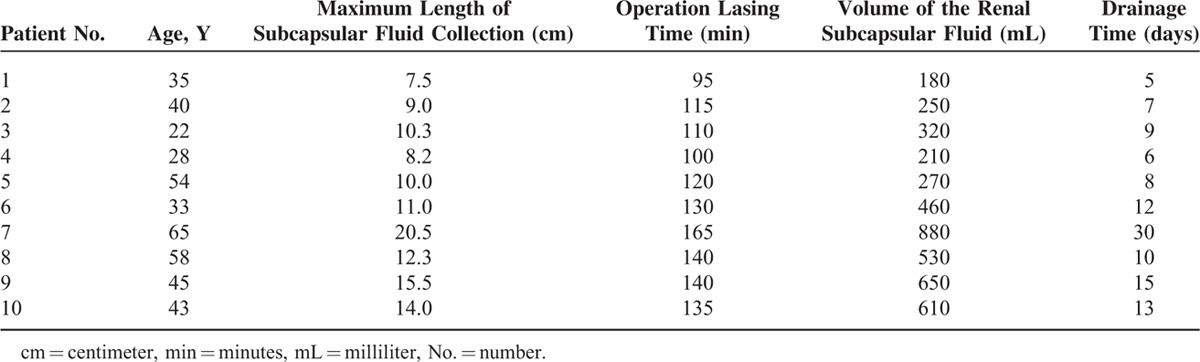
Clinical Parameters of 10 Patients With Spontaneous Renal Subcapsular Fluid Collection

**FIGURE 2:**
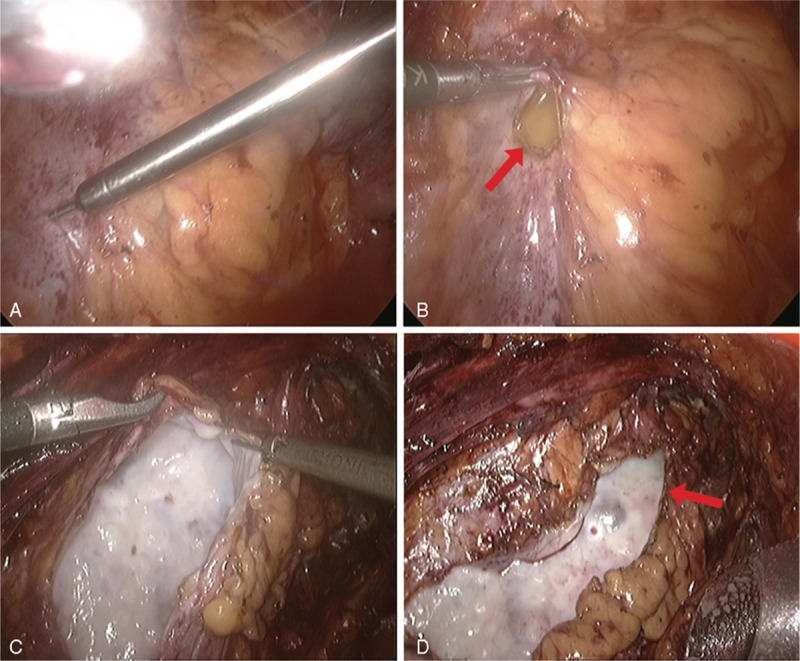
Retroperitoneal laparoscopic renal capsulectomy in a patient with spontaneous renal subcapsular fluid collection. The laparoscopic puncture needle suction was inserted into the surface of the diseased renal capsula (A). The renal capsula was opened by a laparoscopic hook electrode, and renal subcapsular fluid of light yellow color could be observed (see arrow, B). The renal capsule was further incised by the laparoscopic ultrasonic dissector (C), and the pale renal parenchyma could be observed from the opened capsula (see arrow, D).

**FIGURE 3:**
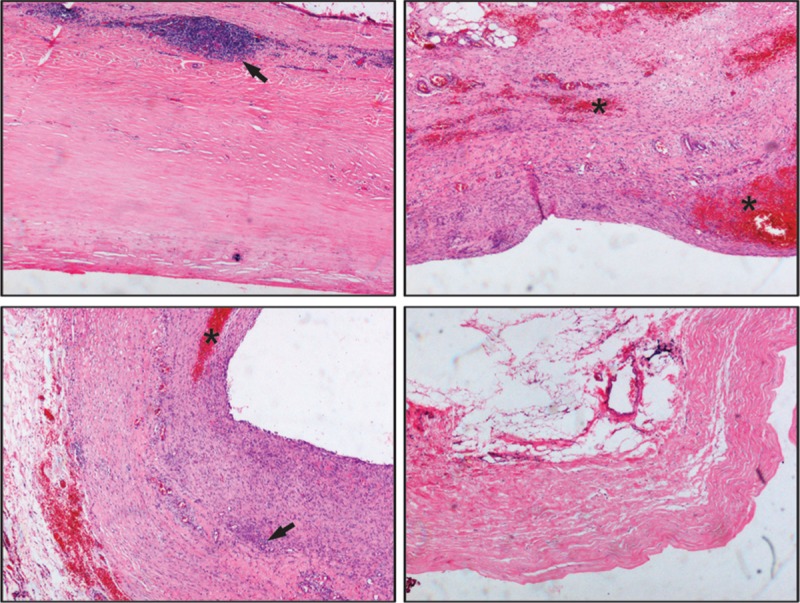
The 4 representative pathological images of excised renal capsula were examined by hematoxylin-eosin staining with ×40 magnification. Fibrous dysplasia of the renal capsula could be observed with infiltration by inflammatory cells (see arrows) together with old hemorrhage foci in some areas (see stars).

Retroperitoneal laparoscopic renal capsulectomy was successfully performed in all 10 patients with no major complications. The mean operation time was 125 min (95–165 min, Table 2). During the surgery, a thickened renal capsula could be observed with severe adhesions to the perirenal fat. Renal subcapsular fluid with a light yellow color was sucked out by the puncture needle suction, and the renal capsula was opened, unroofed, and excised by a laparoscopic hook electrode or a laparoscopic ultrasonic dissector (Figure 2). The average volume of the renal subcapsular fluid was 436 mL (180–880 mL, Table 2) with a mean concentration of creatinine and urea nitrogen of 59.5 micromole per liter (μmol/L, 38–90 μmol/L, Table 3) and 4.81 millimole per liter (mmol/L, 3.07–6.88 mmol/L, Table 3), respectively, which is quite similar to serum. The pathological examination indicated fibrous dysplasia of the renal capsula and infiltratration by inflammatory cells, with old hemorrhage foci in some areas (Figure 3).

**TABLE 3:**
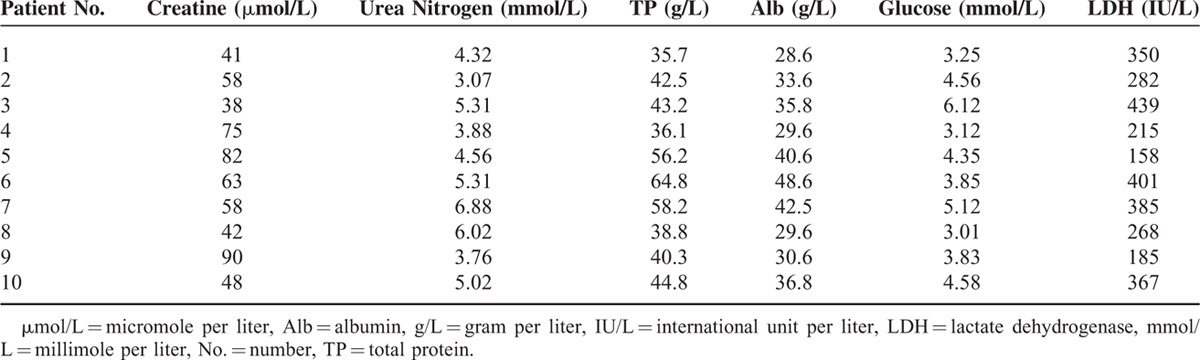
Biochemical Test Results of Renal Subcapsular Fluid From 10 Patients With Spontaneous Renal Subcapsular Fluid Collection

The average time for drainage after the operation was 11.5 days (5–30 days, Table 2). Patient #7 had the largest volume of renal subcapsular fluid collection in this cohort and the relatively largest wound after the operation, thus with the longest postoperative drainage time of 30 days. All the patients completely recovered and had no flank or abdominal pain 1 month after the operation. Renal subcapsular fluid collection nearly ceased and no recurrence could be detected with a mean follow-up period of 12 months (6–24 months, Figure 1).

Ten publications involved patients with renal subcapsular fluid, were found from the PubMed online database together with one of our previous published articles in Chinese from our institute were analyzed, and some key data were extracted and summarized in Table 1. Most of them were clinical case reports with only 1 case. Among those 11 publications, only 2 papers reported 11 patients with spontaneous renal subcapsular fluid collection; however, the other 9 papers presented 23 patients with secondary renal subcapsular fluid collection. According to the literature, the management strategies for those totally 34 patients included conservative treatment, ultrasound-guided percutaneous drainage, and renal capsulectomy. To be more specific, conservative treatment should first be suggested for patients with small amount or asymptomatic fluid collection; the ultrasound-guided percutaneous drainage should be recommended for patients with unbearable lumbar pain or infective complications and less effective to conservative therapy, and the open or laparoscopic renal capsulectomy should be performed to patients who were refractory to percutaneous drainage.

## DISSCUSSION

Kidneys, including the renal collecting system, are highly vascular organs and the major fluid gateway of the body. They are surrounded by a fibrous renal capsule that is very easily detached from the renal parenchyma. Additionally, the kidneys and ureters are adjacent to the abdominal organs, so the renal pelvis and ureters can be easily affected by abdominal organ diseases. These anatomical spaces are the basic structures for the formation of renal subcapsular fluid collection,^4^ which may be infrequently seen in the clinic.

The content of renal subcapsular fluid may include blood, pus, urine, transudate, and lymphatic fluid. Examination of the aspirate fluid is an essential method for specifying the kind of renal subcapsular fluid collected. Analysis of fluid composition is also helpful for identifying urine leakage, because higher creatinine and potassium concentrations and lower sodium concentrations are detected in urine than in hematomas, abscesses, and lymphocele fluid. Indeed, urinary fistulae can be responsible for renal subcapsular fluid collection, particularly through postsurgical ureteral or bladder leaks. Renal subcapsular hematomas can occur during the postoperative period or after trauma and are easily diagnosed by examining the fluid composition. Abscesses are also easily identified by aspirate white blood cell composition and bacterial cultures.^5,6^

The common etiologies of renal subcapsular fluid collections include renal cell carcinoma, renal vascular diseases, hemorrhagic renal cyst, spontaneous calyceal rupture, hydronephrosis, preeclampsia, renal trauma, iatrogenic injury, upper urinary obstruction, and blood dyscrasia; however, the pathophysiological mechanism for the formation of spontaneous renal subcapsular fluid collection is still unknown.^7^ It might be caused by enhanced leakage from renal capillaries due to increased capillary permeability through capillarectasia^8^; additionally, its relationship to allergic disease is still inconclusive.^9^ Liu et al suggested that it might be related to the rupture of proximal convoluted tubule cysts or fistulas, as they reported 1 case with left kidney massive subcapsular fluid collection, which was refractory to percutaneous drainage with the continuous transduate at the daily mean volume of 1064 mL within the follow-up of 52 days. Finally, the patient received an open renal capsulectomy with the naked kidney parenchyma covered by the greater omentum, which had the great absorption power to make internal drainage to completely cure the disease. During the surgery, the author observed many needlepoint-sized cysts on the surface of kidney parenchyma under the excised renal capsula, and some transduate fluid could be identified continuously from those tiny cysts. The authors considered that those tiny cysts might arise from the renal tubules and contained glomerular filtrate or epithelial secretion, and could contain crude urine through extracellular matrix remodeling. Due to the expended volume of crude urine in those individual cysts, the fistula of proximal convoluted tubule could be formed, causing the persistent leakage of the glomerular filtrate and ultimately contributing to the formation of renal subcapsular fluid collection.^10^ However, in our cohort of patients, we did not observe those needlepoint-sized cysts on the surface of kidney parenchyma under the excised renal capsula in the operation, so whether the rupture of proximal convoluted tubule cysts or fistulas plays a key role in the formation of spontaneous renal subcapsular fluid collection is still controversial.

Interestingly, accordingly to the literature, many of the patients with renal subcapsular fluid collection are female (20 females to 14 males, data shown in Table 1), and our cohort also contained 10 female patients with spontaneous renal subcapsular fluid collection, making the female to male ratio as 30 to 14. Most importantly, the ratio for patients with spontaneous renal subcapsular fluid collection was 20 females to 1 male. So, we suspect that some unknown self-immune disorders, which contribute to the formation of the renal subcapsular fluid, might exist in these female patients, but the complicated mechanism of it cannot be specified due to the limited number of patients. Further clinical research should be undertaken with larger number of patients to elucidate possible sex differences in the incidence of this disease.

Patients with renal subcapsular fluid collection usually do not have specific symptoms, but most of them may have flank or abdominal pain, and some may have hematuria or flank lump. Symptoms like fever or hypertension may be observed, and physical examination may find flank percussion pain or abdominal tenderness.^11,12^ The diagnosis mainly relies on B ultrasound, CT scan, and magnetic resonance imaging, which all show crescent images with liquid density under the renal capsula, or a higher density if they are hemorrhagic lesions. Imaging should exclude flank trauma, renal cell carcinoma, renal cyst, renal vascular diseases, kidney inflammation, hematoma, and urinary obstruction.^13^ Obviously, B ultrasound is an easy and prompt way to diagnosis patients with renal subcapsular fluid collection; however, it can neither determine the character of the collected fluid, nor clearly display the outline of the kidney.^14^ Contrast CT scan is the most comprehensive diagnostic modality both for the diagnosis and evaluation of the patients with renal subcapsular fluid collection.^15^ In our cohort study, all the patients were diagnosed by contrast CT scan, which showed crescent images with liquid density but no enhancement under the renal capsula.

The etiologies and pathophysiological mechanisms for the formation of spontaneous renal subcapsular fluid collection are still unknown, and the treatment strategies are mainly focused on relieving symptoms. The conservative managements include antibiotics, control of pain or high blood pressure with monitoring of vital signs should first be suggested for the patients with renal subcapsular fluid, especially for those with small amount or asymptomatic fluid collection. However, if it seems less effective for more than 2 weeks, even presenting with unbearable lumbar pain or infective complications, then the ultrasound-guided percutaneous drainage should be recommended for prevention of kidney function impairment and a potential secondary hypertension. Although it seems very effective for most patients, its efficacy may be limited for some patients. If the daily drainage volume is above 500 mL and lasting for more than 2 weeks with no obvious symptom relief, or if the volume of renal subcapsular fluid remains with no obvious reduction or accumulated again identified by ultrasonography, then those patients should be recommended to receive open or laparoscopic renal capsulectomy, as performed in our cohort of the patients.^16^ The key points for this surgery are to remove the renal capsula of the diseased area as much as possible and to eliminate the secretory capacity of the renal capsula.^17^ The possible mechanism for this successful surgery is to stop the continuous fluid collection by increasing the pressure between the kidney and perirenal fat and fascia; the fluid might be directly absorbed by the perirenal and retroperitoneal fat tissues after the surgery.

It is very interesting to notice that Hao et al also reported 10 female patients presenting spontaneous renal subcapsular fluid collection, among them, 8 patients were successfully treated with ultrasonography-guided drainage of the collection; however, in our patients cohort, 8 patients with the same disease could not be treated by percutaneous drainage. After carefully reading the published article, we actually found that the authors did not mention the size of the renal subcapsular fluid collection of those 8 patients, only presenting an representative CT scan image with the maximum length of subcapsular fluid collection of the right kidney around 7 cm, according to the scale bar.^18^ However, in the cohort of our patients, the mean maximum length of renal subcapsular fluid collection was 11.8 cm (7.5–20.5 cm), which were much bigger than the size of fluid collection in the published cohort, and could not be effectively treated by percutaneous drainage. Eventually, they were completely recovered through retroperitoneal laparoscopic renal capsulectomy. We believe that the size difference of fluid collection between the published cohort and our patient cohort might make this discrepancy for the outcome of the percutaneous drainage. It may indicate again that the ultrasonography-guided drainage should be recommended to newly diagnosed patients, and renal capsulectomy can be considered for patients with recurrent disease or who were refractory to drainage therapy.

## CONCLUSION

Spontaneous renal subcapsular fluid collection is very rare in the clinic, and its etiology and clinical pathophysiological mechanism is still unknown. The treatment strategy mainly relies on alleviating the symptoms, which may include lumbar pain or high blood pressure. Conservative treatments include antibiotics; control of pain or high blood pressure with monitoring of vital signs should first be suggested especially for patients with small amount or asymptomatic fluid collection. However, if it seems less effective, even presenting with unbearable pain or infective complications, then percutaneous subcapsular renal puncture and drainage should be recommended. Importantly, for some patients with recurrent disease or refractory to drainage therapy, the retroperitoneal laparoscopic renal capsulectomy can be strongly considered with excellent treatment effect. Here, we presented our 10-case experience of patients with spontaneous renal subcapsular fluid collection. These patients underwent retroperitoneal laparoscopic renal capsulectomy, with successful results and no recurrence. Future clinical studies with expanded patient numbers may elucidate the possible pathophysiological mechanisms of this condition.
